# Predictors of Musculoskeletal Pain among Primary School Students Using Smartphones in Nakhon Si Thammarat, Thailand

**DOI:** 10.3390/ijerph191710530

**Published:** 2022-08-24

**Authors:** Jittaporn Mongkonkansai, Siriluk Veerasakul, Shamsul Bahri Mohd Tamrin, Uraiwan Madardam

**Affiliations:** 1 Department of Occupational Health and Safety, School of Public Health, Walailak University, Nakhon Si Thammarat 80161, Thailand; 2 Center of Excellence in Data Science for Health Study, Walailak University, Nakhon Si Thammarat 80161, Thailand; 3 Department of Environmental and Occupational Health, Faculty of Medicine and Health Sciences, Universiti Putra Malaysia, Serdang 43400, Malaysia

**Keywords:** smartphone-usage posture, sitting posture, lying down posture, musculoskeletal pain, primary school student

## Abstract

School-age children increasingly use smartphones to conduct their learning activities; increasing reports of disorders related to smartphone use exist, including visual-related symptoms, stress, and musculoskeletal pain. This study aimed to examine risk factors for musculoskeletal pain among primary school students using smartphones. A cross-sectional study was conducted with 233 school-aged children in Nakhon Si Thammarat, Thailand. Data collection used a questionnaire for musculoskeletal symptoms using the Nordic Musculoskeletal Questionnaire with ISO 11,226:2000. Through Chi-square, *t*-test, and logistic regression analysis, factors independently associated with musculoskeletal pain were determined. An important factor in the development of musculoskeletal pain was the prolonged use of smartphones for longer than 60 min, particularly among children aged 6–9 years old. In regards to musculoskeletal pain, almost 53% of the students used their smartphones while lying down. Posing in a prone position while using a smartphone was 7.37 times more dangerous than sitting. The laying position tilts numerous organs at varying angles, especially the upper arm. The risk of musculoskeletal complaints must be reduced by educating parents, children, and the relevant government organizations about safe smartphone usage. The mentioned factors may be used to anticipate the onset of musculoskeletal pain caused by smartphone use in young children.

## 1. Introduction

Musculoskeletal pain is the most common problem worldwide. This results in severe limitations in mobility and maneuverability [1]. Musculoskeletal pain can be found in all genders and ages, including children. Musculoskeletal pain in adolescence is similar to that in adults [1]. Several studies have reported back pain in the spine, trunk, neck, upper limbs, and lower limbs [1,2,3]. Children above 11 years of age have more musculoskeletal pain than those below 11 [3]. Recently, internet usage among children 6–14 years increased from 96.4% in 2021 to 96.7% in 2022 in Thailand [4]. The screen times of the children’s internet users revealed that 89.97% use the internet 5–7 days per week, and most children spend 1–2 h using the device per day [4]. Parents provided their children with devices when the former did household chores (70%) to keep the latter distracted and calm (65%). In Philadelphia, USA, almost all the children (96.6%) use mobile devices and most started using the devices before 1 year old [5]. A previous study demonstrated smartphone addiction in children [6].

A major problem with the musculoskeletal system among children addicted to smartphones exists, and they may develop faulty habitual posture due to constant neck flexion, which may even place them at high risk of spine abnormalities [7]. The report showed flawed flexion of neck and back in children using smartphones [7], resulting in a prevalence of musculoskeletal pain that was substantially higher among school adolescents [3,8]. Neck pain in children and adolescents has also been associated with use of smartphones and tablets [7]. The children bent their backs while using smartphones in the range of 8.50–8.54 degrees and reclined backward by 12–24 degrees while viewing the screen [9]. Both of these ranges are dangerous concerning back and neck injuries. Hansraj KK. found that flexing the neck by 60 degrees while using the phone is the same as applying a 60-pound weight to the spine [10]. Although several studies have focused on the use of smartphones in sitting positions for adolescents and adults [5,7,8,11], a dearth of factual investigation exists on the use of smartphones among school-aged children, especially in lying down positions [12]. Lying down posture had the maximum physical movement while using a smartphone, followed by the sitting posture, which created a lot of movement, intensifying the ergonomic risk [12]. A lack of information exists concerning risk factors for the development of musculoskeletal pain among primary school students. Researchers have conducted a relatively small number of incidence studies on musculoskeletal pain in children. The information gathered through our rigorous experiments can prospectively be used to prevent the occurrence of musculoskeletal disorders and signs of musculoskeletal symptoms due to smartphone use among school-aged children. This study examined smartphone-associated risk factors for musculoskeletal pain among primary school students.

## 2. Materials and Methods

### 2.1. Design

This research was a cross-sectional survey study. The study was conducted between May 2019 and April 2020 on primary school students in Nakhon Si Thammarat province, Thailand (8°25′33.2″ N 99°57′47.6″ E).

### 2.2. Participants

The researcher chose a primary school in the Nakhon Sri Thammarat Province with permission from the school director. Inclusion criteria included school students between grades 1–6; use of smartphones for at least 6 months; and attended school on the day of data collection. The study recruited 233 students with permission from their parents or caregiver. The sample groups were separated into six groups using a phased sampling procedure, each subdivided into a classroom. The proportions of the sample groups were then computed. Then the total number of samples in each classroom was calculated. A simple random sample of all students in the classroom was selected.

### 2.3. Research Instrument

The questionnaires were evaluated for tool quality assessment with a conformance value of Index of Item–Objective Congruence (IOC) equal to 0.9. They showed strong validity and information regarding musculoskeletal pain using Cronbach’s alpha of 0.83.

The instruments used comprise the following four parts:

Part 1: General information about gender, age, education level, medical condition, exercise, surgery history, and hobbies are in this essential information questionnaire.

Part 2: Information on smartphone use is as follows: smartphone ownership, smartphone usage duration (in years), screen size, daily usage period, smartphone usage frequency, parents’ smartphone usage rules, and smartphone usage postures are all addressed.

Part 3: The musculoskeletal pain was investigated using a body discomfort map and a modified Nordic Musculoskeletal Questionnaire [13], separating the organs into four parts: head, trunk, shoulders and upper arms, and lower arms. The students were asked to color the afflicted region on the map to reflect the musculoskeletal pain they were experiencing. The pain was classified into five categories over the previous 3 months: never, once a month, once a week, more than once per week, and frequently a week. Once, we asked the kids directly about the last 3 months. We then transmitted the questionnaire replies to the parents of each student in order to confirm the accuracy of the information. Following that, the data were used to examine the consequences. Based on a World Health Organization survey of children’s health behavior, students with symptoms more than once per week and musculoskeletal disorders in at least one body area were determined to have musculoskeletal diseases [13].

Part 4: The students’ smartphone-usage postures were evaluated using a goniometer to measure the movement angle of four body parts, including the head/neck, trunk, upper arm, and lower arm, while using the smartphone in various positions, including sitting and lying down, and compared to the reference criteria from ISO 11,226:2000 Ergonomics-Evaluation of static working postures [14]. In the sitting position, a straight line through the body point (reference posture) is used as a reference, and in the sleeping position, the supine or lying down position is employed as a reference. The difference in the angle of the reference posture and the angle of the body part while using the smartphone in different poses causes body part inclination.

The researcher established a space ideal for using smartphones in students’ daily lives, complete with seats, sofas, mats, cushions, and other items, where the students demonstrated various smartphone postures. For each bodily component, the reference criteria included two levels of interpretation: acceptable and unacceptable. The researcher interviewed them and adjusted the difficulty level of the questions to understand them better by using pictures, and, in some instances, additional information was acquired from parents, as depicted in Table S1 and Figure S1.

### 2.4. Data Collection

The researcher interviewed students having permission from their parents and primary school instructors to participate in the study. They conducted a face-to-face interview in a conference room established by the school. Each person’s interview took about 10–15 min. The researcher established the appropriate equipment for students to be as close to the equipment used daily to measure posture. Then, the researchers took photos and video recordings for 5 min to estimate the risk posture. The pupils next demonstrated how to operate the smartphone normally, sitting, and lying down.

### 2.5. Data Analysis

This study uses descriptive statistics, including frequency, percentage, mean, standard deviation, maximum, and minimum (Table 1).

A *t*-test was used to examine different methods of body part angle in a posture with individuals experiencing musculoskeletal pain while using a smartphone (Table 2). Moreover, Chi-squared tests were used to examine the association between characteristic data and relationships between independent variables and musculoskeletal pain (Table 3 and Table 4). IBM SPSS version 28.0 was used to conduct all the tests. The significance level was set up to *p* < 0.05.

### 2.6. Ethical Approval

The human research ethics committee of Walailak University approved the study. (Approval number: WUEC-20-131-01). The researcher communicated the study objectives and content to the school instructors and parents to obtain their permission to conduct research among the pupils. In addition, after describing the project to students, the researchers obtained written informed permission from students who volunteered to participate in the study.

## 3. Results

### 3.1. Characteristic Data

The majority of students (64.80%) were female, and 63.09% were 10–12 years old. The majority of students (91.84%) had no underlying diseases and had been exercising (83.27%), and owned a smartphone (79.83%). The smartphone’s screen size was less than 5 inches (approximately 14 cm). Students devoted more than 60 min on their smartphones per day (70.81%). The duration (in minutes per day) of smartphone use increased with age. The age group with the longest length of smartphone use was 12 years. The large majority of parents have rules about smartphone usage (59.22%). The results suggest that sitting (47.21%) is the most common position for school-aged children to adopt while using a smartphone (Table 1).

**Table 1:** Characteristic Data.

Characteristic Data	*n*	%
Gender		
Male	82	35.20
Female	151	64.80
Age		
6–9 years	86	36.91
10–12 years	147	63.09
Underlying disease		
Do not have	214	91.84
Have	19	8.16
Exercising		
Do not exercise	30	16.73
Exercise	203	83.27
Owning a smartphone		
No	47	20.17
Yes	186	79.83
Smartphone screen size		
<6 inches	196	84.13
≥6 inches	37	15.87
Smartphone usage duration (minutes per day)		
<60 min	68	29.19
≥60 min	165	70.81
Smartphone usage duration by Age (minutes per day)		
6 yrs (Mean ± SD = 91.25 ± 32.24)	4	1.72
7 yrs (Mean ± SD = 77.37 ± 51.30)	19	8.15
8 yrs (Mean ± SD = 83.52 ± 35.27)	27	11.59
9 yrs (Mean ± SD = 127.97 ± 80.08)	36	15.45
10 yrs (Mean ± SD = 147.76 ± 108.21)	49	21.03
11 yrs (Mean ± SD = 152.16 ± 104.73)	44	18.88
12 yrs (Mean ± SD = 166.59 ± 113.19)	54	23.18
Parents’ smartphone usage rules		
Do not have	95	40.78
Have	138	59.22
Posture		
Sitting	110	47.21
Supine	84	36.05
Prone	39	16.74

### 3.2. Comparison between the Different Means of Body Part Angle in Posture Having Musculoskeletal Symptoms While Using a Smartphone

The average head/neck angle in sitting posture was found to be the largest (*p* < 0.001) when body part angles were compared between sitting and lying down (supine and prone posture). The average trunk and upper arm angles were the largest in the prone position (*p* < 0.001) (Table 2).

**Table 2:** Comparison between the different mean of body part angle in posture.

Factor	*n* (%)	Mean Angle (Degree)	*p*
Head/Neck			
Sitting	110 (47.21)	24.38	<0.001 **
Supine	84 (36.05)	8.57	
Prone	39 (16.74)	18.97	
Trunk			
Sitting	110 (47.21)	11.85	<0.001 **
Supine	84 (36.05)	4.88	
Prone	39 (16.74)	19.87	
Upper arm			
Sitting	110 (47.21)	17.64	<0.001 **
Supine	84 (36.05)	24.77	
Prone	39 (16.74)	78.33	
Lower arm			0.547
Sitting	110 (47.21)	63.83	
Supine	84 (36.05)	63.75	
Prone	39 (16.74)	62.84	

Statistical significance was conducted using *t*-test ** *p* < 0.001.

### 3.3. Relationships between Factors and Ages

Smartphone ownership, parental smartphone usage rules, and self-care after feeling discomfort/fatigue were statistically significantly (*p* < 0.05) associated with age. Overall, 79.83% of the participants owned a smartphone, and most were less than 10 years old. The duration of smartphone use was for more than 60 min daily in 70.82% of them, and most were younger than 10. Children 10–12 years (43.35%) engaged more in self-care after experiencing discomfort or fatigue than those younger than 10 (Table 3).

**Table 3:** Relationships between factors and ages.

Characteristic Data	Age. *N* (%)		*p*
	6–9 years	10–12 years	
Owning a smartphone			<0.001 **
No	31 (13.30)	16 (6.87)	
Yes	55 (23.61)	131 (56.22)	
Smartphone usage duration (minutes/day)			0.008 *
<60 min	34 (14.59)	34 (14.59)	
≥60 min	52 (22.32)	113 (48.50)	
Self-care after experiencing discomfort or fatigue			<0.001 **
No	60 (25.75)	46 (19.74)	
Yes	26 (14.59)	101 (43.35)	
Parents’ smartphone usage rules			0.796
No	36 (15.41)	59 (25.32)	
Yes	50 (21.50)	88 (37.77)	

Statistical significance was performed using Chi-squared tests * *p* < 0.05, ** *p* < 0.001.

### 3.4. Logistic Regression Analysis of Musculoskeletal Symptoms

Table 4 shows that age, owning a smartphone, parents’ smartphone usage rules, smartphone usage duration, and posture were significantly (*p* < 0.05) associated with musculoskeletal symptoms. The OR for musculoskeletal symptoms increased by 10.31-fold in smartphone usage duration more than 60 min/day (95% CI: 4.18, 25.46, *p* < 0.001), by 7.37-fold in prone posture (95% CI: 2.68, 20.31, *p* < 0.001), by 7.39-fold in parents’ smartphone usage rules (95% CI: 3.51, 15.59, *p* < 0.001), and by 4.04-fold in age group 6–9 years (95% CI: 1.78, 9.18, *p* = 0.001). The equation for logistic regression was as follows:

Logit(P) = −5.421 + 0.749 _(Gender)_ + 1.394 _(Age)_ + 1.171 _(Owning smartphone)_ − 2.333 _(Smartphone usage duration)_ + 2.001 _(Parents’ smartphone usage rules)_ + 1.737 _(Supine Posture)_ + 1.998 _(Prone Posture)._

**Table 4:** Logistic regression analysis for musculoskeletal symptoms.

Factor	Crude OR			Adjusted OR		
	OR	95%	*p*-Value	OR	95%	*p*-Value
Gender						
Male	1	-	-	1	-	-
Female	1.72	1.00–2.97	0.050	2.11	0.99–4.54	0.054
Age						
10–12 years	1	-	-	1	-	-
6–9 years	1.33	0.78–2.26	0.301	4.04	1.78–9.18	0.001 *
Owning a smartphone						
No	1	-	-	1	-	-
Yes	2.59	1.32–5.10	0.006	3.23	1.28–8.13	0.013 *
Parent’s smartphone usage rules						
Yes	1	-	-	1	-	-
No	6.47	3.59–11.66	<0.001	7.39	3.51–15.59	<0.001 **
Smartphone usage duration						
<60 min	1	-	-	1	-	-
≥60 min	8.17	4.06–16.44	<0.001	10.31	4.18–25.46	<0.001 **
Posture						
Sitting	1	-	-	1	-	-
Supine	1.120	0.51–2.48	0.780	5.68	2.55–12.67	<0.001 **
Prone	0.272	0.13–0.59	0.001	7.37	2.68–20.31	<0.001 **

Adjusted by gender, age, owning a smartphone, parent’s smartphone usage rules, smartphone usage duration, posture. OR= Odd ratio, 1= reference value. Statistical significance was performed using logistic regression analysis * p < 0.05, ** p < 0.001.

## 4. Discussion

The time spent using a smartphone was the best predictor of musculoskeletal discomfort among school students between grade 1 to 6. Students using their smartphones for more than 60 min daily are 10 times more likely to develop musculoskeletal disorders (MSDs) than those who use a smartphone for less than 60 min daily. It has been reported that prolonged use of smartphones without parental rules is associated with depression and sleep time in children [15]. Sleep problems have been shown to be a risk factor for onset of musculoskeletal pain in adolescents, while in children it is not clear yet [16]. Students using their smartphones for more than 2 h daily were more likely to have lower back, neck, and shoulder pain [17]. While texting on a touchscreen smartphone, around half of the participants had neck and shoulder discomfort and weariness [18,19]. Students using smartphones for more than 3 h daily are more likely to have upper back discomfort than those spending less than an hour with them. The more time spent on smartphones, the more painful body parts were observed.

Posture while using smartphones in the lying position especially prone posture was associated with a higher risk of MSDs than sitting. The body posture with the greatest total movement was lying; next, sitting; and finally, standing [12]. The results of the previous study suggest that standing or sitting at a desk is necessary for properly using a computer, tablet, or smartphone [20]. Moreover, we found that lying posture is significantly associated with MSDs and similar findings were reported among primary children and university students with smartphone usage [21,22]. The lying down posture produced the most physical movement, followed by the sitting posture, with a lot of movement increasing the ergonomic risk with symptoms in the upper arm (10%) and lower arm (5%). When using a smartphone lying, the mean upper arm angle was larger than in the sitting position (sitting = 17.64, supine = 24.77° and prone = 78.33°). The subject’s shoulders are raised in the lying position to help the users use the smartphone. The elbow is supported in the supine position, and the arm angle may be adjusted to bring the smartphone closer to eye level. Long periods of raising the shoulders and elbows can be exhausting [12]. Consequently, the neck angle induced by lying down is smaller than that generated by sitting. In the current study, the average head and body flexion/bending among students were 30 and 20 degrees, suggesting an average body angle of 25.6 degrees and maximum back flexion/extension of 50 degrees with smartphone usage [9]. When looking at the head angle, about half of the students stooped their heads in the sitting and lying down positions. However, using a smartphone while sitting does not provide full head support and increases the risk of musculoskeletal injury. With neck flexion, the weight placed on the head bones rises. In the neutral posture, a fully developed head weighs 5 kg. The weight on the neck increases when the head bends forward, reaching 18 kg at 30 degrees and 27 kg at 60 degrees [10]. Although the typical head and trunk angles are within the acceptable limit, students’ smartphone usage can last up to 40 min and more than 2 h daily, indicating a risk of musculoskeletal complaints.

Students with no smartphone regulations (40.78%) experienced seven times higher musculoskeletal pain than those with a smartphone rule because children could use their smartphones as they would like. If parents do not enforce stringent limits regarding their children’s smartphone usage, it may harm their health [23]. However, parental control may not prevent smartphone addiction [15]. Limiting smartphone usage may result in academic degradation [24]. Proactive management is preferred, and parents should discuss the advantages and disadvantages of smartphone use with their children [2]. MSD risk was higher in children under 10 than in those over 10. According to Thor-burn, E. et al., symptoms are likely to increase at a particular level of device usage after raising in frequency. The majority of symptomatic participants immediately felt sick within the first 30 min of use [25]. Children under the age of 10 exhibited less self-care after experiencing discomfort or fatigue than children over the age of 10. They did not take a break as they were using their smartphones continuously. Furthermore, Keya F.D.s revealed that the 8.5-year-old case study had been playing the game all day. Even while eating and sleeping, she held her mobile phone in her hand [26]. Therefore, if young children used smartphones without taking breaks, they were more likely to become fatigued. For school students, prolonged smartphone use should not exceed 10 min to minimize biomechanical effects in the neck area [27]. However, the trend of students using smartphones illustrated a continuously increasing pattern [28]. Therefore, the protection of the student’s health is critical. Students, parents, school administrators, and all the relevant government agencies need to earnestly focus on the problem of students’ smartphone usage to prevent musculoskeletal symptoms at a younger age.

## 5. Conclusions

Age, owning a smartphone, smartphone usage duration, parents’ smartphone usage rules, and posture were associated with musculoskeletal symptoms. Using smartphones with a prone posture presented a higher risk of developing ergonomic-related musculoskeletal complaints. Those using their smartphones for more than 60 min daily are 10 times more likely to develop MSDs than students using them for less than 60 min. According to the parents’ smartphone usage rules, students with no smartphone regulations experienced six times higher musculoskeletal pain than those with a smartphone rule.

## 6. Suggestions for Students and Parents

1. Parents should have rules on using smartphones and restrict the usage time to less than 60 min daily. Parents should not encourage children to own smartphones.
2. School-aged children using smartphones should use a chair or sofa to support the head, neck, body, and shoulders, and avoid using smartphones in the lying-down position.
3. Parents and teachers should guide and educate students concerning proper phone usage posture, including smartphone usage benefits and drawbacks.

## Data Availability

The datasets generated during and/or analyzed during the current study are available from the corresponding author upon reasonable request.

## Supplementary Materials

The following supporting information can be downloaded at: https://www.mdpi.com/article/10.3390/ijerph191710530/s1. Figure S1: The illustration of the body’s inclination during reference posture and smartphone usage. A represents sitting posture, B represents supine posture and C represents prone posture; Table S1: Static Postural Assessment.
