# Supplementary material for: Predictors of Musculoskeletal Pain among Primary School Students Using Smartphones in Nakhon Si Thammarat, Thailand
Source: Int J Environ Res Public Health. 2022 Aug 24;19(17):10530. doi: 10.3390/ijerph191710530 (PMC9518098; doi:10.3390/ijerph191710530)
Supplement: Supplementary file 1 [file ijerph-19-10530-s001.zip › ijerph-1859368-supplementary.pdf]

Figure S1: The illustration of the body's inclination during reference posture and smartphone usage. A represents sitting posture, B represents supine posture and C represents prone posture.

A. Sitting Posture

1. Head (Reference posture)<br>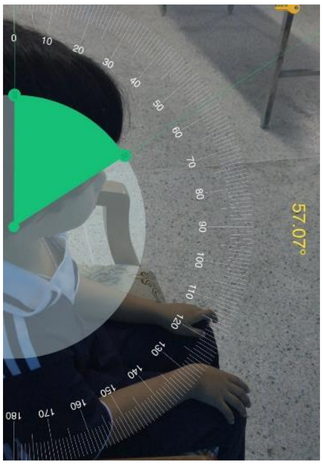 	1. Head (Smartphone usage posture)<br>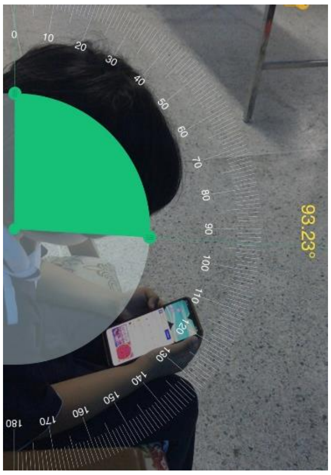
2. Trunk (Reference posture)<br>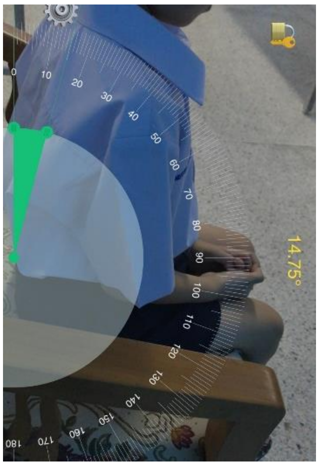 	2. Trunk (Smartphone usage posture)<br>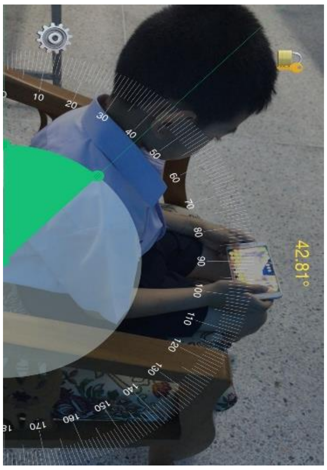
3. Upper arm (Reference posture)<br>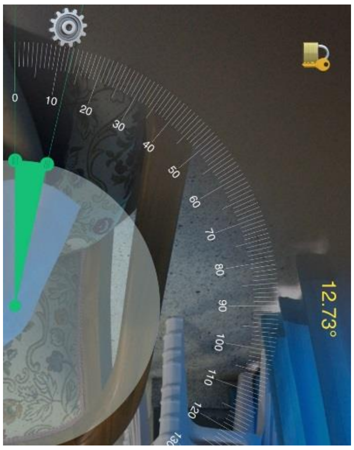 	3. Upper arm (Smartphone usage posture)<br>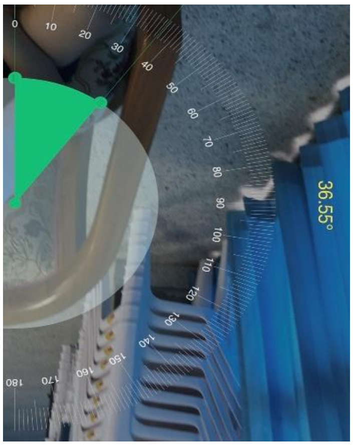

A. Sitting Posture

4. Lower arm (Reference posture)	4 .Lower arm (Smartphone usage posture)
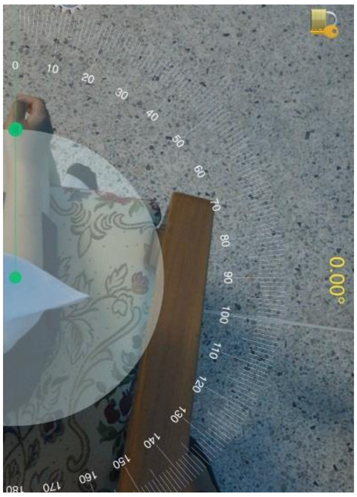	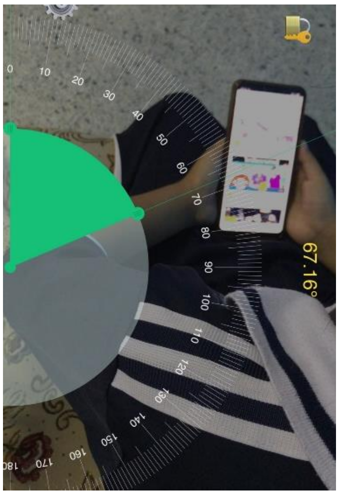
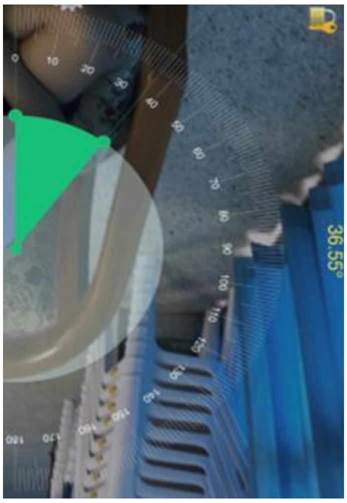	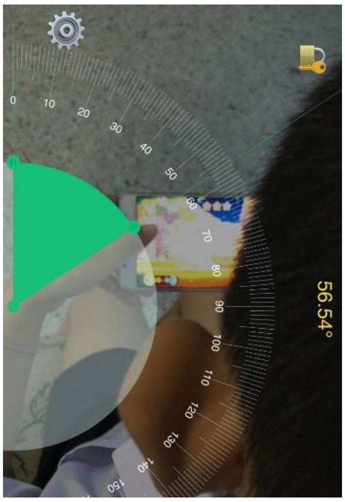

B. Lying down posture (Supine position)

1.Head (Reference posture) 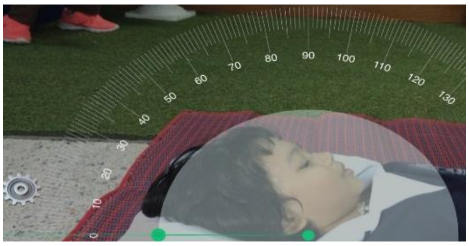	1.Head (Smartphone usage posture) 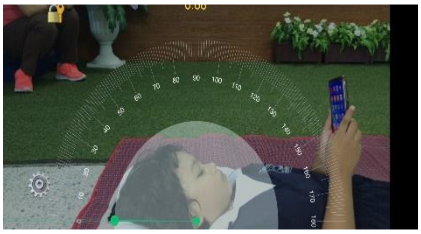
2. Trunk (Reference posture) 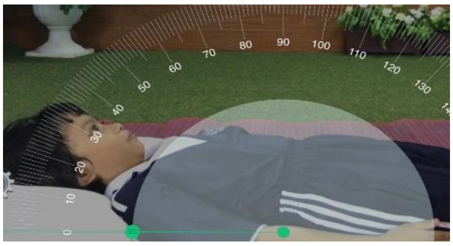	2. Trunk (Smartphone usage posture) 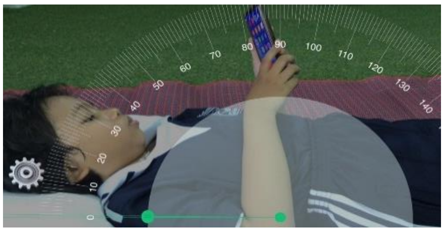
3. Upper arm (Reference posture) 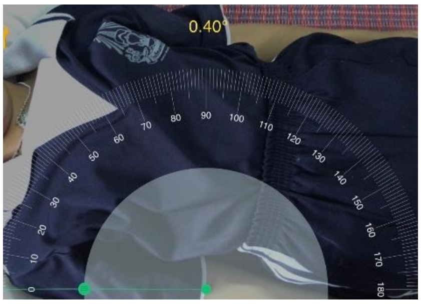	3. Upper arm (Smartphone usage posture) 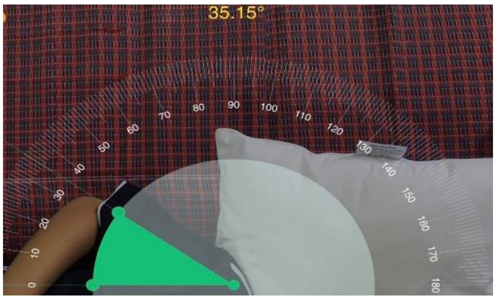
4.Lower arm (Reference posture) 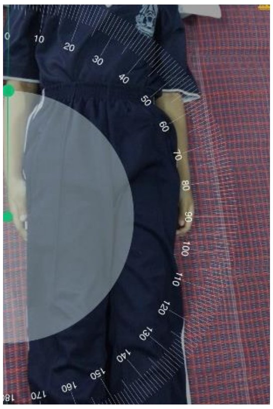 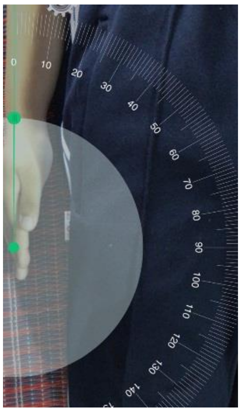	4.Lower arm (Smartphone usage posture) 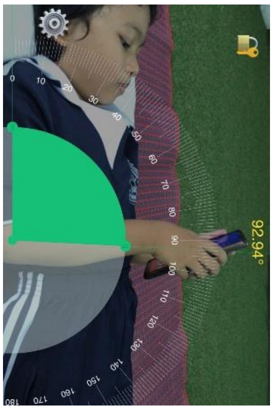 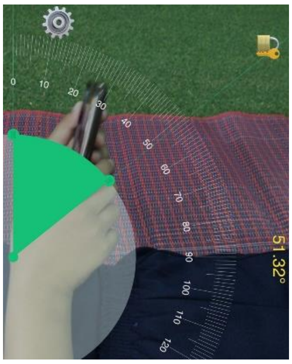

C. Lying down posture (Prone position)

1.Head (Reference posture)	1.Head (Smartphone usage posture)
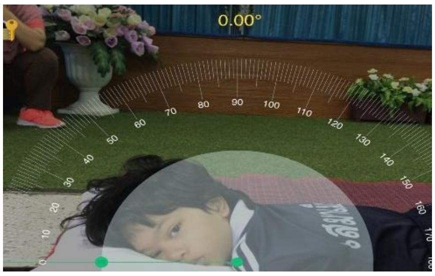	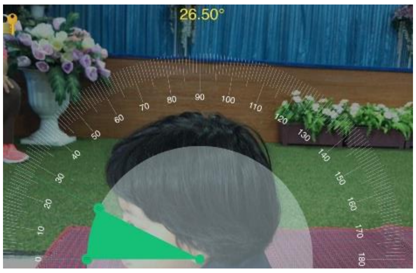
2. Trunk (Reference posture) 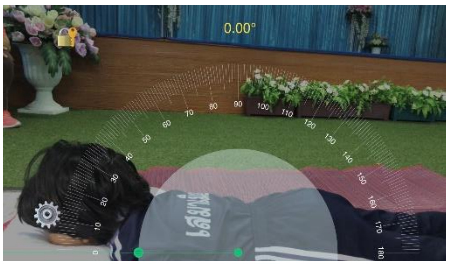	2. Trunk (Smartphone usage posture) 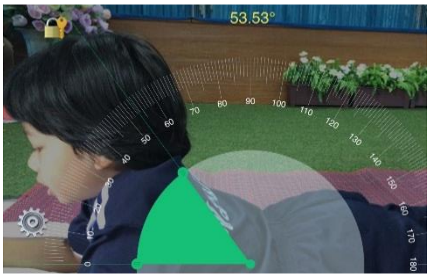
3. Upper arm (Reference posture) 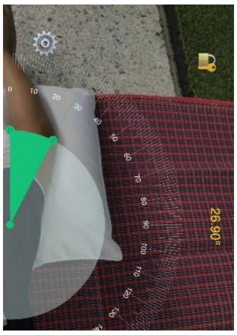	3. Upper arm (Smartphone usage posture) 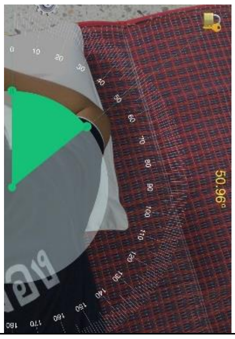
4.Lower arm (Reference posture) 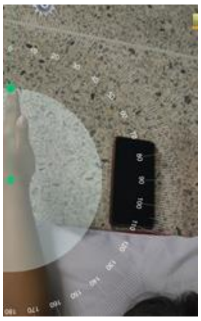 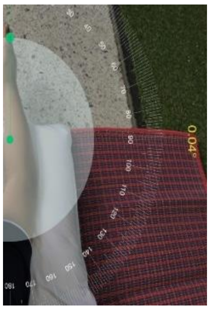	4.Lower arm (Smartphone usage posture) 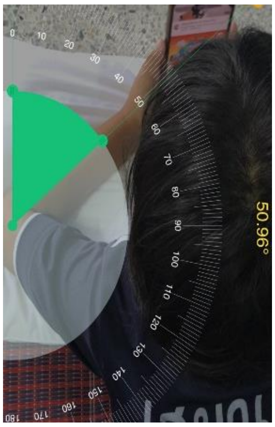 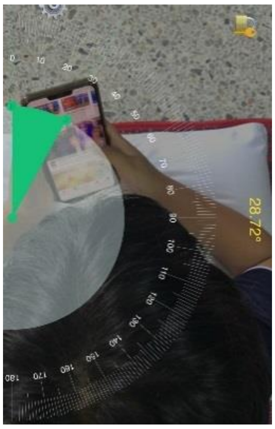

Table S1: Static Postural Assessment

Body Part Inclination	Head Inclination	Trunk Inclination	Upper Arms	Lower Arms
**Reference posture**	The angle between lobe of the ear and lateral cortex of the eye	The angle between the upper edge of the greater trochanter and spinous process of the seventh cervical vertebra	The angle between the acromioclavicular joint and the humeroradial joint	Natural position
**Acceptable**	1. Symmetrical posture<br>2. < 0° with full head support<br>3. 0°–20°<br>4. 25°–85° with full trunk support and holding time for head inclination at the setting value<br>5. 25°–85° without full trunk support and neck flexion/extension 0°–20°	1. Symmetrical posture<br>2. < 0° with full trunk support<br>3. 0°–20°<br>4. 20°–60° with full trunk support	1.No awkward upper arm posture<br>2. 0°–60° with full arm support<br>3. 20°–60° without full arm support and holding time for upper arm elevation at the setting value	1. Extreme elbow (flexion ≤ 150°/ extension > 10°<br>2. Extreme forearm pronation ≤ 90°/ supination ≤ 60°<br>3. Extreme wrist posture ≤ 20°<br>: radial abduction ≤ 30°<br>: ulnar abduction ≤ 90°<br>: flexion<br>: extension ≤ 90°
**Unacceptable**	1. < 0° without full head support<br>2. 25°–85° with full trunk support and holding time for head inclination > the setting value<br>3. 25°–85° without head trunk support and neck flexion/extension > 25°<br>4. > 85°	1. < 0° without full trunk support<br>2. 20°–60° without full trunk support and holding time for upper arm elevation > the setting value<br>3. > 60°	1. Awkward upper arm posture<br>2.raised shoulder<br>3.20°–60° without full arm support and holding time for upper arm elevation at the setting value<br>4. > 60°	1. Extreme elbow (flexion > 150°/ extension > 10°<br>2. Extreme forearm pronation 90°/ supination 60°<br>3. Extreme wrist posture 20°<br>: radial abduction 30°<br>: ulnar abduction 90°<br>: flexion<br>: extension 90°
